# Clinical Efficacy and Safety of Human Mesenchymal Stem Cell Therapy for Degenerative Disc Disease: A Systematic Review and Meta-Analysis of Randomized Controlled Trials

**DOI:** 10.1155/2021/9149315

**Published:** 2021-09-13

**Authors:** Baocheng Xie, Shichun Chen, Yongxiang Xu, Weichao Han, Runkai Hu, Minyi Chen, Ruirong He, Shaobo Ding

**Affiliations:** Department of Pharmacy, Affiliated Dongguan Hospital, Southern Medical University, Dongguan, Guangdong, China

## Abstract

Degenerative disc disease (DDD) can cause severe low back pain, which will have a serious negative impact on the ability to perform daily tasks or activities. For the past few years, mesenchymal stem cell (MSC) transplantation has emerged as a promising strategy for the treatment of DDD. However, the clinical efficacy of MSC in the treatment of DDD still lacks clinical evidence and is controversial. We conducted a meta-analysis with randomized controlled trials (RCTs) to evaluate the clinical efficacy and safety of MSC transplantation in patients with DDD. We searched major databases using terms from the database's inception through March 2021. The Cochrane bias risk assessment tool was used to assess quality. The analysis showed that MSC therapy could decrease visual analog scale (VAS) scores (SMD = −0.50, 95%CI = −0.68 ~ −0.33, *P* < 0.00001) and Oswestry Disability Index (ODI) scores (SMD = −0.27, 95%CI = −0.44 ~ −0.09, *P* = 0.003). The outcomes with subgroup analysis showed that MSC therapy could decrease VAS scores in 3 months (*P* = 0.001), 6 months (*P* = 0.01), 12 months (*P* = 0.02), and ≥24 months (*P* = 0.002) and ODI scores in ≥24 months (*P* = 0.006). Pooled analysis showed that MSC therapy has a higher ratio of patients at most thresholds but particularly at the MIC (minimally important change) (*P* = 0.0002) and CSC (clinically significant change) (*P* = 0.0002) in VAS and MIC (*P* = 0.0005) and CSC (*P* = 0.001) pain responders in ODI. Adverse events (AE) of treatment-emergent adverse events (TEAE), back pain, arthralgia, and muscle spasms were not statistically significant between the two groups. However, our further statistical analysis showed that MSC therapy may induce AE of TEAE related to study treatment (OR = 3.05, 95%CI = 1.11 ~ 8.40, *P* = 0.03). In conclusion, this study pooled the main outcomes and showed that MSC therapy could significantly decrease VAS and ODI scores in patients with DDD. Distinctly, the findings of this meta-analysis suggest a novel therapeutic strategy for patients with chronic low back pain (LBP) and lumbar dysfunction by DDD.

## 1. Introduction

Degenerative disc disease (DDD) is a multifaceted, progressive, and irreversible disease. It is an inescapable part of aging and may lead to a series of diseases or symptoms such as lumbar disc herniation, cervical spondylosis, discogenic pain, spinal stenosis, and spinal segment instability [1, 2]. DDD often results in severe LBP that would have a severe negative impact on the ability to perform daily tasks or activities [3]. This has developed into a public health problem that seriously affects the socioeconomic and quality of life of the people [4]. For patients who have failed conservative treatments (nonsteroidal anti-inflammatory drugs (NSAIDs), nonpharmacologic treatment with superficial heat, physical therapy, chiropractic, and/or acupuncture), it is particularly important to find new, safe, and effective treatment strategies [5]. Currently, the surgical treatment options for DDD mainly include laser, nucleus pulpoplasty, interbody fusion, and artificial disc replacement. Although these treatments have achieved good short-term results, long-term outcomes are affected by the high probability of recurrent pain. There is literature regarding the poor efficacy of spine fusion for treating LBP [6]. In addition, none of the previously mentioned treatment regimens could alleviate or reverse the course of disc degeneration. Disc removal could also lead to further deterioration of the degeneration due to spinal instability [7, 8].

Therefore, the focus of current research on DDD is increasingly turning to regenerative methods to slow down continuous degenerative cascade. The degenerative disc microenvironment must stabilize in order to achieve this goal and eventually return to a normal physiological state. So, the potential therapeutic strategies include (a) reversing protein expression of proinflammatory cytokines/proteinases in intervertebral disc cells, (b) blocking proinflammatory cytokines/proteases in extracellular matrix, (c) producing new extracellular matrix in intervertebral disc cells by intervention, and (d) addition of additional cells to support regeneration in a degenerative intervertebral disc environment [9]. One promising therapeutic strategy for inducing intervertebral disc regeneration may be the use of progenitor cells and stem cells [10]. In recent years, the use of MSC for intervertebral disc regeneration has been most widely studied [10]. MSC has excellent immune privilege and immune evasion and suppresses ongoing immune response in a way that is not restricted by the human leukocyte antigen system [11, 12]. In the first place, we found that many animal studies of mesenchymal stem cell transplantation for DDD are particularly promising. Yim et al. [13] included 24 animal studies on MSC transplantation for DDD. All three types of mesenchymal stem cells have certain advantages in inhibiting intervertebral disc degeneration. To sum up, evidence suggested that MSC transplantation could increase disc space height in animal models. Bracingly, Orozco et al. [14] studied the injection of bone marrow MSC in 10 patients diagnosed with DDD and chronic back pain. After MSC transplantation treatment, 85% of patients had significantly reduced lumbar pain and disability in 3 months. After 6 and 12 months, a significant increase in the water content of the patient's intervertebral discs was observed, with moderate improvements [14]. Meanwhile, Noriega et al. [15] conducted a RCT in which 24 patients had been diagnosed with DDD. In the MSC group, bone marrow MSC were injected into each intervertebral disc. Results showed that the pain and disability of patients were significantly reduced at 3 months after MSC transplantation. Of greatest concern, the results of the study found no difference in VAS scores between the MSC transplantation treatment group and the control group at 12 months. Another RCT result also found that after MSC+allograft treatment, VAS score and ODI score decreased compared to baseline, but there was no statistical difference in 3, 6, and 12 months, compared to the control group with standard graft material [16]. The clinical efficacy of MSC transplantation in the treatment of DDD remains controversial. Recently, Noriega et al. [17] published long-term 42-month follow-up results of the RCT showing good differences after MSC treatment. Our team believes that for patients with DDD, choosing MSC transplantation is a positive effect on improving patients' VAS and ODI. However, there is currently no high-quality evidence-based medicine to support. In order to further explore the efficacy and safety of MSC transplantation in the treatment of DDD, we conducted this meta-analysis of published RCTs to study the therapeutic evidence of human MSC transplantation for DDD.

## 2. Materials and Methods

The detailed protocol for this study was designed in accordance with the Cochrane intervention review. The entire project has been registered on the PROSPERO website (CRD42021248707). The whole design and writing process of this meta-analysis were done one-to-one according to the of PRISMA.

### 2.1. Literature Search

We searched major databases including PubMed, Embase, and ClinicalTrials.gov using terms from the database's inception through March 2021. We screened the literature based on the participants, interventions, comparisons, outcomes, and study (PICOS) approach. The terms included the following: (1) degenerative disc disease, DDD, intervertebral disc repair, intervertebral disc degeneration, lumbar disc degeneration, and disc degeneration; (2) mesenchymal stem cell and allogeneic mesenchymal precursor cells; and (3) randomized controlled trials.

### 2.2. Extraction of Study Data

Two data extractors (He RR and Chen SC) screened the full text of MSC in the treatment of DDD and extracted the main observation indicators. The extraction of experimental data is mainly filled in the data extraction form table designed in advance. Disputable data were resolved through a third independent investigator (Xu YX). The main aspects of data extraction were registration, number of participants, age, treatment strategy, duration, and observed outcome.

### 2.3. Risk Assessment of Bias in Included Studies

To further address the risk of bias between included studies, we use the Cochrane risk of bias tool to assess the quality of the literature one by one for the included studies.

### 2.4. Outcome Indicators

(1) The efficacy outcomes are as follows: visual analog scale (VAS) of 3, 6, and 12 months or ≥24 months and Oswestry Disability Index (ODI) of 3, 6, and 12 months or ≥24 months. (2) The other efficacy outcomes are as follows: MIC and CSC of 6 and 12 months or ≥24 months. (3) The safety outcomes are as follows: adverse events (AE) of MSC therapy for DDD.

### 2.5. Inclusion and Exclusion Criteria

The inclusion criteria are as follows: (1) RCTs of studies; (2) participants with DDD; (3) the MSC group received MSC treatment and the control group received HA or rehabilitation treatment; and (4) follow-up time was longer than 3 months. Exclusion criteria are as follows: (1) nonrandomized trials; (2) ongoing RCTs without outcomes; (3) review, systematic review, or meta-analysis; and (4) case reports, prospective, or retrospective cohort studies.

### 2.6. Data Synthesis and Analysis

In this study, Review Manager 5.3 and Stata 12.0 were used to conduct statistics and analysis on the data of multiple outcomes, respectively. If the outcomes were dichotomous data, we analyzed the data by odds ratio (OR) and 95% confidence intervals (CIs). The contiguous data to be merged were represented by a standardized mean difference (SMD) and 95% CI. The *χ*^2^ test and *I*^2^ statistic were used to calculate the heterogeneity among the studies. The quantitative value of *I*^2^ < 25% indicated mild inconsistency between the studies, and *I*^2^ that ranged from 25% to 50% indicated moderate heterogeneity. If the *I*^2^ > 50%, this indicates that the study has serious heterogeneity. We would conservatively use the random-effect model to evaluate the statistical significance of the pooled outcomes, so as to reduce the influence of heterogeneity of this study. If *I*^2^ < 50%, the fixed-effect model was used for analysis. Subgroup analyses was used to study effect size of VAS of 3, 6, and 12 months or ≥24 months and ODI of 3, 6, and 12 months or ≥24 months.

## 3. Results

### 3.1. Screening of Studies

**Figure 1:**
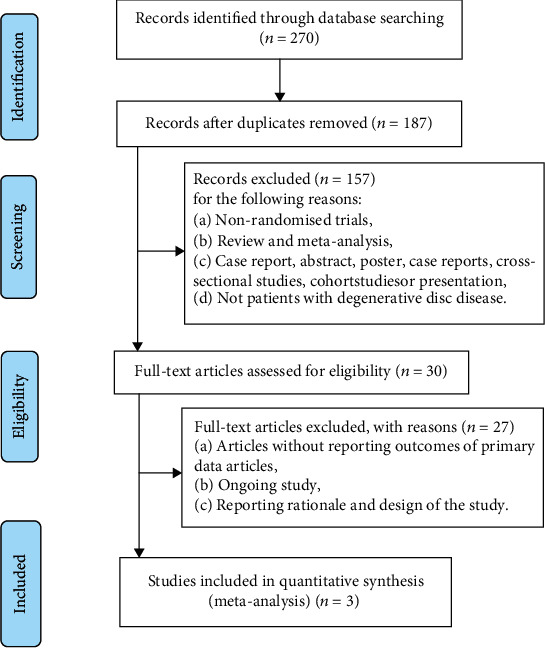
Flowchart of the retrieval strategy.

In this study, through the systematic retrieval of major databases, a total of 270 citations were retrieved from the establishment of the database to March 2021. 83 duplicate studies were excluded after importing the retrieved literature into NoteExpress. After reading the abstracts, 157 studies were excluded for several reasons: (a) nonrandomized trials; (b) systematic review or review; and (c) case reports, cross-sectional studies, and prospective or retrospective cohort studies. Next, after reading the full text, we excluded 27 ongoing studies and studies that were only basic principles and designs. In the end, we included three studies on MSC transplantation in the treatment of DDD for analysis (Figure 1).

### 3.2. Characteristics of Each Study

After screening of the inclusion criteria, 3 studies [15, 17, 18] with 104 participants were finally included in the analysis. The MSC group was treated with MSC, and the control group was treat with HA or mepivacaine. The registration numbers of two RCTs were NCT01290367 and NCT01860417. The MSC therapy was used in three RCTs that were allogeneic mesenchymal precursor cells and allogeneic mesenchymal stem cells. The amount of MSC transplants in the study of Amirdelfan et al. [18] was 6 × 10^6^ and 1.8 × 10^7^. The dosage of MSC transplants in the other RCT was 2.50 × 10^7^. The efficacy outcomes were VAS of 3, 6, and 12 months or ≥24 months and ODI of 3, 6, and 12 months or ≥24 months. The other efficacy outcomes were MIC and CSC of 6 and 12 months or ≥24 months. The safety outcomes were AE of MSC therapy for DDD (Table 1).

### 3.3. Quality Assessment of Study

**Figure 2:**
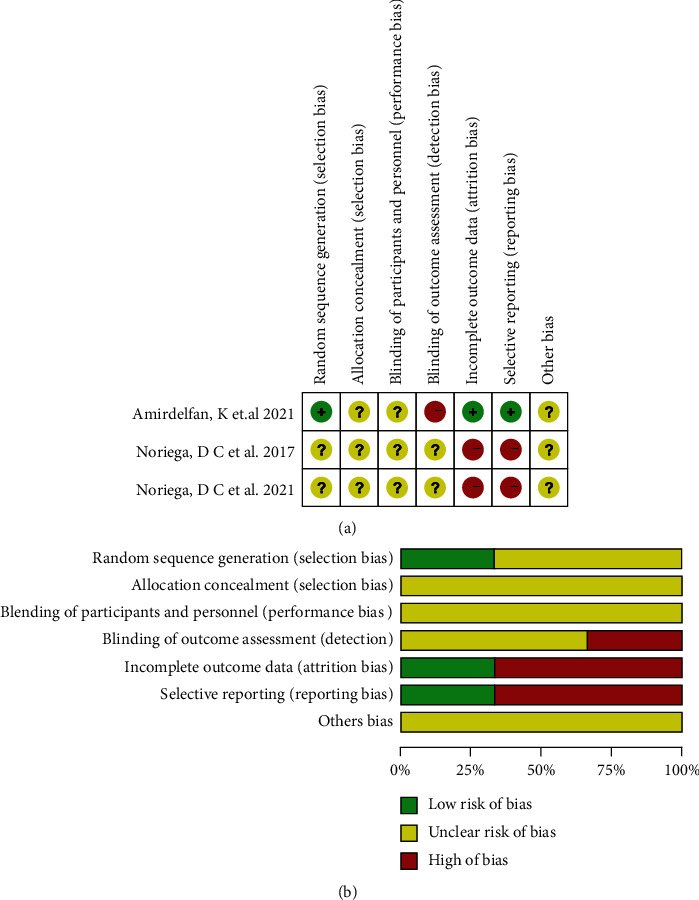
Quality evaluation of studies by the Cochrane collaboration manual. (a) Item-by-item detailed analysis of the summary of the risk of bias in studies. (b) The risk bias graph shows the quality summary of the study.

The clinical trials of Amirdelfan et al. [18] were divided into two groups according to a central randomization schedule and randomization list. In terms of selection bias, we assessed them as “low risk” studies. The study of Noriega et al. [15] did not explicitly address the randomized approach, which we assessed as “unclear” of selection bias. The clinical trial of Amirdelfan et al. [18] reported that the participants and radiographic reviewer were blinded to the assigned treatment but the investigator was not blinded. So, we assessed it as “unclear risk” in selection bias and performance bias. The trial of Noriega et al. [15] reported that the participant, care provider, and outcome assessor were blinded, but we did not find the specific implementation plan for blinding, so we assessed it as “unclear risk” in detection bias, selection bias, and performance bias. Amirdelfan et al. [18] fully reported the outcome measures including the number of people lost to follow-up and the reasons for dropping out. We assessed it as “low risk of bias” in attrition bias and reporting bias. The clinical trial of Noriega et al. [15] did not report the number of people lost to follow-up or dropped out, so we assessed it as “high risk of bias” in attrition bias and reporting bias (Figure 2).

### 3.4. Visual Analog Scale

**Figure 3:**
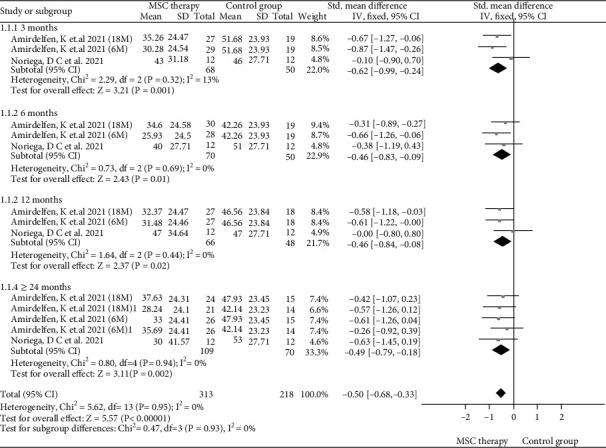
Forest plot of VAS scores between MSC therapy and control group.

VAS was reported in three studies [15, 17, 18] of MSC therapy and control group. We used a fixed-effect model to evaluate the statistical significance of the pooled analysis after testing for heterogeneity (*I*^2^ = 0 < 50%). The result of a meta-analysis showed that MSC therapy could significantly decrease VAS scores (SMD = −0.50, 95%CI = −0.68 ~ −0.33, *P* < 0.00001), compared with the control group. Subgroup analysis of VAS scores is as follows: the result with a fixed-effect model showed that MSC therapy could significantly decrease VAS scores in 3 months (SMD = −0.62, 95%CI = −0.99 ~ −0.24, *P* = 0.001), 6 months (SMD = −0.46, 95%CI = −0.83 ~ −0.09, *P* = 0.01), 12 months (SMD = −0.46, 95%CI = −0.84 ~ −0.08, *P* = 0.02), and ≥24 months (SMD = −0.49, 95%CI = −0.79 ~ −0.18, *P* = 0.002) (Figure 3).

### 3.5. MIC and CSC Responders in VAS

We used a fixed-effect model to evaluate the statistical significance of MIC and CSC responders in VAS of patients. Pooled analysis showed that MSC therapy has a high ratio of patients at most thresholds, especially in MIC (change ≥ 30% from baseline) (OR = 2.16, 95%CI = 1.43 ~ 3.25, *P* = 0.0002) and CSC (change ≥ 50% from baseline) (OR = 2.18, 95%CI = 1.44 ~ 3.31, *P* = 0.0002) thresholds. The pain responder rates of MIC and CSC for 2 groups in 6, 12, and ≥24 months are showed in Table 2.

### 3.6. Oswestry Disability Index

**Figure 4:**
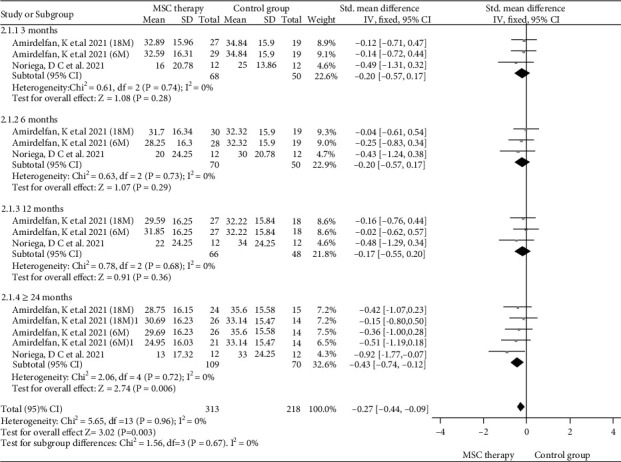
Forest plot of ODI scores between MSC therapy and control group.

ODI scores were reported in three studies [15, 17, 18] of MSC therapy and control group. A fixed-effect model was used with heterogeneity analysis (*I*^2^ = 0%). The result of the meta-analysis showed that MSC therapy could decrease ODI scores (SMD = −0.27, 95%CI = −0.44 ~ −0.09, *P* = 0.003) (Figure 4). Subgroup analysis of ODI is as follows: the result with the fixed-effect model found that MSC therapy could significantly decrease ODI scores in ≥24 months (SMD = −0.43, 95%CI = −0.74 ~ −0.12, *P* = 0.006) in patients with DDD (Figure 4). However, no statistical differences were found in the subgroup analysis in 3, 6, and 12 months.

### 3.7. MIC and CSC Pain Responders in ODI

We used a fixed-effect model to evaluate the statistical significance of MIC and CSC pain responders in ODI of patients. Pooled analysis showed that MSC therapy has a high ratio of patients at most thresholds, especially in MIC (change ≥ 10 − point ODI from baseline) (OR = 2.06, 95%CI = 1.37 ~ 3.10, *P* = 0.0005) and CSC (change ≥ 15 − point ODI from baseline) (OR = 2.01, 95%CI = 1.33 ~ 3.05, *P* = 0.001) thresholds. The pain responder rates of MIC and CSC in 6, 12, and ≥24 months are showed in Table 2.

### 3.8. Adverse Event

To further clarify the safety of MSC transplantation for DDD, meta-analysis was performed on the occurrence of AE. The result showed that AE of treatment-emergent adverse events (TEAE) (OR = 1.11, 95%CI = 0.40 ~ 3.07, *P* = 0.84), back pain (OR = 1.23, 95%CI = 0.55 ~ 2.76, *P* = 0.62), arthralgia (OR = 0.63, 95%CI = 0.19 ~ 2.11, *P* = 0.45), and muscle spasms (OR = 2.11, 95%CI = 0.40 ~ 11.01, *P* = 0.38) were not statistically significant between two groups. However, our further statistical analysis showed that MSC therapy may induce AE of TEAE related to study treatment (OR = 3.05, 95%CI = 1.11 ~ 8.40, *P* = 0.03) (Table 3).

## 4. Discussion

### 4.1. Primary Efficacy Outcomes

Pain assessment is the prerequisite for pain treatment for chronic LBP by DDD. Accurate and timely assessment of pain can provide necessary guidance and assistance for clinical treatment and is the key to effective pain treatment [19]. The VAS is the most commonly used scoring index in pain assessment [20]. The specific practice of VAS is to draw a horizontal line of 10 cm on the paper, and one end of the horizontal line is 0, indicating painless; the other end is 10, which means severe pain; and the middle part represents different levels of pain. The specific clinical assessment was as follows: (1)*Mild pain (1-3 points)*: the patient had pain but tolerable, lived a normal life, and had no disturbance in sleep. (2) *Moderate pain (4-6 points)*: the pain is obvious and intolerable, the patient is required to take analgesic drugs, and his sleep is disturbed. (3) *Severe pain (7-10 points)*: severe pain, unbearable pain, need to use analgesic drugs, severe sleep disturbance, autonomic nervous disorder, or passive posture. Kumar et al. [21] performed a trial of 10 eligible DDD patients. The result showed that primary efficacy outcomes of VAS for low back pain were significantly reduced in 1 month, 3 months, and 6 months after adipose tissue-derived MSC transplantation. The RCT of Noriega et al. [15] showed that VAS scores were significantly reduced at 3 months and 6 months after MSC transplantation. Of greatest concern, the study found that VAS scores increased at 12 months after MSC transplantation. Recently, Noriega et al. [17] published long-term 42-month follow-up results of the RCT. The result showed that MSC therapy could significantly decrease VAS scores at 42 months, compared with the control group in patients with DDD. In this study, we integrated the results of three RCTs, and the results showed that MSC therapy could significantly decrease VAS scores (*P* < 0.00001). A subgroup analysis of VAS scores was used for analysis. The result showed that MSC therapy could significantly decrease VAS scores in 3 months (*P* = 0.001), 6 months (*P* = 0.01), 12 months (*P* = 0.02), and ≥24 months (*P* = 0.002) in patients with DDD. Our study with a small sample size also found that MSC therapy has a high ratio of patients at most thresholds, especially in MIC (*P* = 0.0002) and CSC (*P* = 0.0002) thresholds. Our research results indicated that MSC therapy has shown excellent efficacy in reducing the VAS score of patients with DDD, whether it is short-term treatment or long-term follow-up.

ODI can accurately and reliably assess the treatment effect of patients with chronic LBP and the lumbar dysfunction by DDD [22]. ODI is composed of 10 questions, including the intensity of self-care, lifting objects, pain, sitting, standing, walking, disturbing sleep, social activities, sex life, and travel. There are 6 options for each question, and the maximum score for each question is 5 points. The higher the score, the more severe the patient's dysfunction [23]. Pang et al. [24] studied the feasibility and safety of MSC transplantation for patients with chronic discogenic LBP. After 2 years of follow-up, it was found that after MSC transplantation, the intervertebral disc pain was alleviated, and the ODI score was also significantly reduced. The RCTs of Noriega et al. [15, 17] showed that ODI scores were significantly reduced at 3, 6, 12, and 42 months after MSC transplantation. The results suggest that MSC transplantation is a potential alternative for the treatment of chronic discogenic LBP. In our study, ODI scores were reported in four studies of MSC therapy and control group. Pooled analysis indicated that MSC therapy could significantly decrease ODI scores (*P* = 0.003). Subgroup analysis found that MSC therapy could significantly decrease ODI scores in ≥24 months (*P* = 0.006) in patients with DDD. However, there was no statistically significant difference between 3, 6, and 12 months, but the ODI score had a tendency to decrease, which we believe may be caused by the insufficient sample size of the included studies. We evaluated the statistical significance of MIC and CSC pain responders in ODI of patients. Pooled analysis showed that MSC therapy has a high ratio of patients at most thresholds, especially in MIC (*P* = 0.0005) and CSC (*P* = 0.001) thresholds. To sum up, the results of this meta-analysis suggest that MSC transplantation could significantly alleviate pain and functional degradation in patients with DDD.

### 4.2. Primary Safety Outcomes

MSC has strong attractiveness and application prospects due to their low immunogenicity, easy access, and immunosuppressive potential [25–27], but the safety of MSC is still the first priority. Studies have shown that the quality of MSC depends more on the isolation conditions, cell culture technology, age of the donor, genetic characteristics, and medical history between different donors [28–30]. The quality difference of MSC is closely related to the AE in patients. To further clarify the safety of MSC transplantation for DDD, a meta-analysis was performed on the occurrence of AE. The result showed that AE of TEAE (*P* = 0.84), back pain (*P* = 0.62), arthralgia (*P* = 0.45), and muscle spasms (*P* = 0.38) was not statistically significant between two groups. However, our further statistical analysis showed that MSC therapy may induce AE of TEAE related to study treatment (*P* = 0.03). This study showed that there were no differences in serious AE of MSC transplantation for patient with DDD, compared with the control group. However, it is vital to pay close attention to whether the AE of patients is directly related to MSC therapy.

### 4.3. Limitations and Publication Bias

We found that there was mild inconsistency between the studies in VAS and ODI scores. We analyzed the sensitivity of VAS and ODI scores. The analysis showed that the conclusion was credible, and there was no substantial change of the results with VAS and ODI scores. However, it should not be ignored that a few studies seriously limited the further analysis of publication bias and heterogeneity. To further address the risk of bias between included RCTs, we evaluated the quality of the literature using the Cochrane risk of bias tool. Our evaluation found that the included studies had a low risk of selection bias of randomization. The study describes the generation method of a random assignment sequence in detail. We believe that the results are reliable and stable to a certain extent, but we cannot rule out publication bias caused by a small sample size.

## 5. Conclusions

This meta-analysis pooled the main outcomes and showed that MSC therapy could significantly decrease VAS and ODI scores in patients with DDD. Distinctly, the findings of this meta-analysis suggest a novel therapeutic strategy for patients with chronic LBP and lumbar dysfunction by DDD. But, what is the optimal dose, frequency, time, and route of MSC transplantation at different stages of DDD? These crucial problems urgently require more randomized controlled trials to solve.

## Figures and Tables

**Table 1:** Characteristics of each study.

Study	Registered number	Design	Participants	Intervention		Dosage	Follow-up	Outcomes
				MSC group	Control group			
Amirdelfan et al. [18] 2021	NCT01290367	RCT	80	MSC+HA	HA	6 × 10^6^, 1.8 × 10^7^	36 m	VAS,ODI, AE, CSC, MIC
Noriega et al. [17] 2021	NCT01860417	RCT	24	MSC	Mepivacaine	2.50 × 10^7^	42 m	VAS, ODI, AE
Noriega et al. [15] 2017	NCT01860417	RCT	24	MSC	Mepivacaine	2.50 × 10^7^	12 m	VAS, ODI, AE

Note: AE: adverse event; CSC: clinically significant change; HA: hyaluronic acid; ODI: Oswestry Disability Index; MIC: minimally important change; MSC: mesenchymal stem cell; RCT: randomized controlled trial; VAS: visual analog scale.

**Table 2:** Subgroup analysis.

Responder analyses	6 months	12 months	24 months	36 months	Overall effect
*Pain responder*					
MIC in VAS	OR = 2.53 [1.10, 5.84], *P* = 0.03	OR = 2.79 [1.22, 6.36], *P* = 0.02	OR = 2.12 [0.93, 4.48], *P* = 0.07	OR = 1.49 [0.67, 3.34], *P* = 0.33	OR = 2.16 [1.43, 3.25], *P* = 0.0002
CSC in VAS	OR = 2.59 [1.14, 5.90], *P* = 0.02	OR = 3.05 [1.31, 7.12], *P* = 0.01	OR = 1.78 [0.76, 4.16], *P* = 0.18	OR = 1.63 [0.71, 3.71], *P* = 0.25	OR = 2.18 [1.44, 3.31], *P* = 0.0002
*ODI responder*					
MIC in ODI	OR = 2.16 [0.95, 4.92], *P* = 0.07	OR = 1.86 [0.84, 4.15], *P* = 0.13	OR = 2.12 [0.93, 4.84], *P* = 0.07	OR = 2.12 [0.93, 4.84], *P* = 0.07	OR = 2.06 [1.37, 3.10], *P* = 0.0005
CSC in ODI	OR = 1.60 [0.71, 3.58], *P* = 0.25	OR = 1.86 [0.82, 4.23], *P* = 0.14	OR = 2.04 [0.88, 4.47], *P* = 0.10	OR = 2.81 [1.17, 6.74], *P* = 0.02	OR = 2.01 [1.33, 3.05], *P* = 0.001

**Table 3:** Adverse event analysis.

AE	OR and 95% CI	*P*
TEAE	OR (1.11), 95% CI (0.40, 3.07)	0.84
TEAE related to study treatment	OR (3.05), 95% CI (1.11, 8.40)	0.03
Back pain	OR (1.23), 95% CI (0.55, 2.76)	0.62
Arthralgia	OR (0.63), 95% CI (0.19, 2.11)	0.45
Muscle spasms	OR (2.11), 95% CI (0.40, 11.01)	0.38

## References

[1] Hemanta D., Jiang X. X., Feng Z. Z., Chen Z. X., Cao Y. W. (2016). Etiology for degenerative disc disease. *Chinese Medical Sciences Journal*.

[2] Wu P. H., Kim H. S., Jang I. T. (2020). Intervertebral disc diseases part 2: a review of the current diagnostic and treatment strategies for intervertebral disc disease. *International journal of molecular sciences*.

[3] Balagué F., Mannion A. F., Pellisé F., Cedraschi C. (2007). Clinical update: low back pain. *Lancet*.

[4] Katz J. N. (2006). Lumbar disc disorders and low-back pain: socioeconomic factors and consequences. *The Journal of Bone and Joint Surgery*.

[5] Qaseem A., Wilt T. J., McLean R. M. (2017). Noninvasive treatments for acute, subacute, and chronic low back pain: a clinical practice guideline from the American College of Physicians. *Annals of Internal Medicine*.

[6] Hedlund R., Johansson C., Hägg O., Fritzell P., Tullberg T., Swedish Lumbar Spine Study Group (2016). The long-term outcome of lumbar fusion in the Swedish lumbar spine study. *The Spine Journal*.

[7] Cui X. D., Li H. T., Zhang W., Zhang L. L., Luo Z. P., Yang H. L. (2018). Mid- to long-term results of total disc replacement for lumbar degenerative disc disease: a systematic review. *Journal of orthopaedic surgery and research*.

[8] Furunes H., Storheim K., Brox J. I. (2017). Total disc replacement versus multidisciplinary rehabilitation in patients with chronic low back pain and degenerative discs: 8-year follow-up of a randomized controlled multicenter trial. *The Spine Journal*.

[9] Clouet J., Fusellier M., Camus A., Le Visage C., Guicheux J. (2019). Intervertebral disc regeneration: from cell therapy to the development of novel bioinspired endogenous repair strategies. *Advanced Drug Delivery Reviews*.

[10] Vadalà G., Ambrosio L., Russo F., Papalia R., Denaro V. (2019). Interaction between mesenchymal stem cells and intervertebral disc microenvironment: from cell therapy to tissue engineering. *Stem Cells International*.

[11] Stagg J., Galipeau J. (2007). Immune plasticity of bone marrow-derived mesenchymal stromal cells. *Bone Marrow-Derived Progenitors*.

[12] Ankrum J. A., Ong J. F., Karp J. M. (2014). Mesenchymal stem cells: immune evasive, not immune privileged. *Nature Biotechnology*.

[13] Yim R. L., Lee J. T., Bow C. H. (2014). A systematic review of the safety and efficacy of mesenchymal stem cells for disc degeneration: insights and future directions for regenerative therapeutics. *Stem Cells and Development*.

[14] Orozco L., Soler R., Morera C., Alberca M., Sánchez A., García-Sancho J. (2011). Intervertebral disc repair by autologous mesenchymal bone marrow cells: a pilot study. *Transplantation*.

[15] Noriega D. C., Ardura F., Hernández-Ramajo R. (2017). Intervertebral disc repair by allogeneic mesenchymal bone marrow cells: a randomized controlled trial. *Transplantation*.

[16] García de Frutos A., González-Tartière P., Coll Bonet R. (2020). Randomized clinical trial: expanded autologous bone marrow mesenchymal cells combined with allogeneic bone tissue, compared with autologous iliac crest graft in lumbar fusion surgery. *The Spine Journal*.

[17] Noriega D. C., Ardura F., Hernández-Ramajo R. (2021). Treatment of degenerative disc disease with allogeneic mesenchymal stem cells: long-term follow-up results. *Transplantation*.

[18] Amirdelfan K., Bae H., McJunkin T. (2021). Allogeneic mesenchymal precursor cells treatment for chronic low back pain associated with degenerative disc disease: a prospective randomized, placebo- controlled 36-month study of safety and efficacy. *The Spine Journal*.

[19] Thong I., Jensen M. P., Miró J., Tan G. (2018). The validity of pain intensity measures: what do the NRS, VAS, VRS, and FPS-R measure?. *Scandinavian Journal of Pain*.

[20] Reed M. D., Van Nostran W. (2014). Assessing pain intensity with the visual analog scale: a plea for uniformity. *Journal of Clinical Pharmacology*.

[21] Kumar H., Ha D. H., Lee E. J. (2017). Safety and tolerability of intradiscal implantation of combined autologous adipose-derived mesenchymal stem cells and hyaluronic acid in patients with chronic discogenic low back pain: 1-year follow-up of a phase I study. *Stem cell research & therapy*.

[22] Sandal D., Jindal R., Gupta S., Garg S. K. (2021). Reliability and validity of Punjabi version of Oswestry Disability Index in patients with mechanical low back pain. *Journal of Clinical Orthopaedics and Trauma*.

[23] Poder T. G., Carrier N. (2021). Predicting SF-6Dv2 utility scores for chronic low back pain using the Oswestry Disability Index and Roland-Morris Disability Questionnaire. *Expert Review of Pharmacoeconomics & Outcomes Research*.

[24] Pang X., Yang H., Peng B. (2014). Human umbilical cord mesenchymal stem cell transplantation for the treatment of chronic discogenic low back pain. *Pain Physician*.

[25] Kim N., Cho S. G. (2016). Overcoming immunoregulatory plasticity of mesenchymal stem cells for accelerated clinical applications. *International Journal of Hematology*.

[26] Choi J. R., Yong K. W., Nam H. Y. (2019). Current status and perspectives of human mesenchymal stem cell therapy. *Stem Cells International*.

[27] Xie B., Luo H., Zhang Y., Wang Q., Zhou C., Xu D. (2018). Autologous stem cell therapy in critical limb ischemia: a meta-analysis of randomized controlled trials. *Stem Cells International*.

[28] Pachón-Peña G., Serena C., Ejarque M. (2016). Obesity determines the immunophenotypic profile and functional characteristics of human mesenchymal stem cells from adipose tissue. *Stem Cells Translational Medicine*.

[29] Lukomska B., Stanaszek L., Zuba-Surma E., Legosz P., Sarzynska S., Drela K. (2019). Challenges and controversies in human mesenchymal stem cell therapy. *Stem Cells International*.

[30] Xie B., Chen M., Hu R., Han W., Ding S. (2020). Therapeutic evidence of human mesenchymal stem cell transplantation for cerebral palsy: a meta-analysis of randomized controlled trials. *Stem Cells International*.

